# Kinetics and Evolution Modeling of Hydrogen-Induced Cracking in Low-Carbon Steel

**DOI:** 10.3390/ma18163813

**Published:** 2025-08-14

**Authors:** Iván Mortera-Bravo, Jorge Luis González-Velázquez, Diego Israel Rívas-López, Manuel Alejandro Beltrán-Zuñiga

**Affiliations:** Instituto Politécnico Nacional, Metallurgy and Materials Department, Escuela Superior de Ingeniería Química e Industrias Extractivas, Mexico City 07738, Mexico; imorbra@gmail.com (I.M.-B.); drivasl@ipn.mx (D.I.R.-L.)

**Keywords:** hydrogen induced cracking, kinetics, fracture mechanisms, computational model

## Abstract

The kinetics and evolution of hydrogen-induced cracking (HIC) were modeled using a theoretical model developed by Gonzalez to calculate the individual crack growth rate and a computational algorithm based on a Poisson distribution to generate the initial spatial distribution of HIC nuclei. Additionally, the Monte Carlo method was used to model the interconnection of individual HIC cracks. The results of the computational model were compared versus experimental results of HIC induced by cathodic charging experiments in low-carbon steel plates. The model was capable of accurately emulating the kinetics of HIC, considering the first stage of nucleation and growth of randomly dispersed individual HIC cracks, followed by a second stage where the individual cracks interconnect with each other to form large cracks that subsequently grow. The study was complemented with the fractographic examination of the HIC cracks to verify if the fracture mechanism is consistent with the crack morphology and propagation mode in the proposed model. The results indicate that HIC propagation occurs by cleavage and quasi-cleavage mechanisms, with crack interconnection by ductile shear tearing, where the driving force for HIC is the accumulated hydrogen pressure within the internal HIC cracks, explaining why the crack growth rates are nearly constant in each stage of HIC growth.

## 1. Introduction

Hydrogen-induced cracking (HIC) is a common, and yet complex, issue in the oil and natural gas industry, particularly in the transportation and processing of hydrocarbons containing hydrogen sulfide (H_2_S) and carbon dioxide (CO_2_), known as sour environments [1,2,3,4,5,6]. The HIC process can be regarded as a nucleation and growth phenomenon, consisting of the progressive accumulation of molecular hydrogen in trapping sites, which act as nuclei for cracks that further grow due to the influx of hydrogen into the crack cavities [7,8,9,10,11,12,13,14]. In sour environments, hydrogen is generated by the corrosion reaction between the steel pipe and H_2_S, along with the effect of sulfur ions that promote the absorption of hydrogen into the metal.

The freshly formed atomic hydrogen diffuses into the steel plate, transforming into molecular gas at specific trapping sites, most commonly nonmetallic inclusions. This creates high pressures that form internal cracks; as hydrogen continues to accumulate in the crack, a macroscopic crack is formed. This process is known as the “pressure mechanism” [15,16,17].

The modeling of HIC kinetics is an ongoing topic in the oil and natural gas industry. It is so debated that the API 579-1/ASME FFS-1 Fitness-For-Service standard—in its latest edition (2025), in paragraph 7.5.1 HIC and SOHI Growth Rates—establishes that “At the present time there is no widely accepted method to predict the growth rate of active HIC” [18]; therefore, the topic is a current issue at both academic and industrial levels. Moreover, there is no widely accepted international document that evaluates the damage by HIC through mathematical models, such as the one presented here.

To address the challenge of modeling HIC growth, several kinetic models have been developed, starting with Brower in 1994 [19]; followed by Krom [20] and, independently, by González [21] in 1997; and later by Traidia in 2012 [22]. A key limitation of these models is that they are primarily effective in predicting HIC kinetics during the early stages of damage evolution, typically within the first 40 h, and are focused on individual crack behavior. However, both laboratory tests and field inspections have shown that HIC can evolve over several hundred or even thousands of hours, during which the coalescence of individual cracks significantly alters the overall kinetics and crack morphology [23,24]. More recently, an empirical model proposed by Entezari [25] has been introduced to better capture HIC growth kinetics in steel pipelines. This model incorporates key experimental parameters, including hydrogen diffusion rates, the spatial distribution of crack nucleation sites, the presence of secondary phases, and ferritic grain boundary characteristics. The predictive capabilities of this model are enhanced through the application of statistical analysis.

Regarding the role of the microstructure and chemical composition of the steel, it is generally accepted that nonmetallic inclusions (NMIs) are the preferred sites for HIC [26,27,28,29,30]. Consequently, their shape, content, and spatial distribution determine the number and location of the initial HIC cracks, which subsequently influence the morphology and kinetics of HIC [31,32]. Additionally, it has been observed that a high degree of pearlite banding leads to higher HIC growth rates [33,34], while Sojka, J. et al. [35] demonstrated that susceptibility to HIC increases with the manganese enrichment of pearlite bands. Taira et al. [36] further showed that HIC propagates more readily along hard bands of low-temperature transformation microstructures, formed by the segregation of certain alloying elements [14,37]. However, identifying the role of different microstructural constituents in the kinetics of HIC damage remains challenging, although some researchers suggest that this behavior is related to the hydrogen trapping efficiency of the microstructure [3,4,5,6,7,10,11]. Despite these findings, the nucleation and growth of HIC in relation to the microstructure, as well as how the characteristics of preferential nucleation sites affect the kinetics of HIC, are not yet fully understood.

In addition to the previously discussed factors that enhance HIC, other variables, such as medium pH, temperature, grain size, and heat treatment, show a direct influence [14,26,31] on the locations where hydrogen accumulates more easily or diffuses more rapidly, substantially altering the kinetics of HIC. Some of these effects are indirectly incorporated into the proposed numerical simulation model through the spatial distribution of nucleation sites and parameters such as hydrogen flux and the initial size and shape of the cracks.

Although the current model demonstrates a strong predictive capability despite significant simplifications, future versions are expected to enhance both efficiency and accuracy by incorporating additional influencing factors. Accordingly, the aim of this work is to model the statistical nucleation and growth of hydrogen-induced cracking (HIC) in low-carbon steel under cathodic charging conditions, to compare the simulated crack morphology with experimental observations, and to propose a theoretical framework capable of accurately estimating HIC growth rates. This framework will also serve as a basis for future modeling of different nucleation site distributions or secondary phase segregation profiles.

## 2. Materials and Methods

Steel plates measuring 12 cm wide, 18 cm long, and 1.27 cm thick were cut from a new segment of an API 5L X52 steel pipe of 60.96 cm (24 inch) diameter and 1.27 cm (0.500 inch) thickness. The chemical composition of the plate material was determined by optical emission spectrometry (Belec Spektrometrie Opto-Elektronik GmbH, Georgsmarienhütte, Germany), as shown in Table 1.

**Figure 1:**
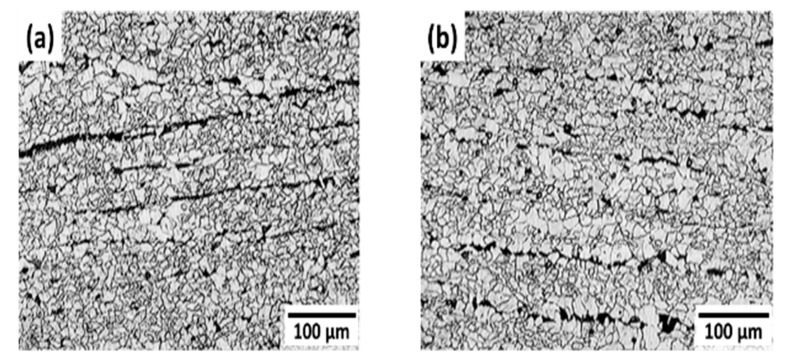
Optical micrograph of API 5L X52 steel: (**a**) longitudinal section and (**b**) transversal section.

Metallographic samples were prepared, sequentially ground up to 600 grit, polished with 1 μm diamond paste, rinsed in double-distilled water, and degreased in acetone. To reveal the microstructure, the samples were chemically etched using Nital solution (10% nitric acid in methanol) and examined under a metallurgical microscope (Olympus, Tokyo, Japan) using the bright field technique. The steel used was produced using an electric arc furnace, followed by secondary metallurgy and continuous casting. The microstructure of the API 5L X52 steel used in the experiment is shown in Figure 1 and consists of a matrix of equiaxed ferrite grains with bands of fine pearlite. The heat treatment condition corresponds to normalization. The average grain size is 20 μm, and the inclusion content was Type A, fine series, according to the standard test method for determining the inclusion content of steel, ASTM E45-18a [38].

The microstructural characterization is presented in Table 2.

### 2.1. Cathodic Charging Procedure

**Figure 2:**
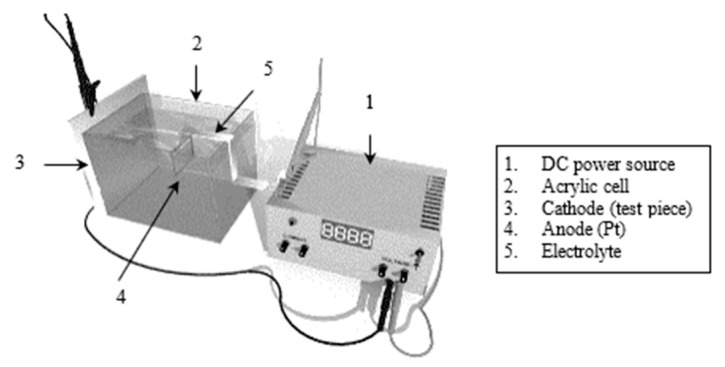
Experimental setup of the cathodic charging system.

The HIC was induced using the cathodic charging method, whose experimental setup is shown in Figure 2. An acrylic cell was glued to one face of the test plate, while the plate was connected as a cathode to a DC power source. An electrolytic solution was poured into the cell, and the circuit was completed with an inert anode. Once the direct electric current was applied, atomic hydrogen was formed on the exposed plate surface and by the effect of a poison added to the electrolyte; the HIC initiation occurred under these conditions.

The surface of the plate to be exposed to the electrolytic solution was ground with abrasive paper up to grade 600 and cleaned by immersion in an ultrasonic bath for 10 min with a commercial cleaning solution. Once the surface was dry and clean, an acrylic cell was glued to one side of the plate and filled with the electrolytic solution, allowing an area of 198 cm^2^ to be exposed to the hydrogen charging. Subsequently, the steel plate was connected as the cathode to an external DC power source. A digital multimeter (Fluke corporation, Everret, WA, USA, connected in series with the electrochemical system, was used to continuously monitor the applied current. Current regulation was also controlled by the DC power source equipment (Sorensen, San Diego, CA, USA), and a platinum grid was used as an anode. Although a detailed statistical analysis of experimental error was not the primary focus in this work, the high degree of control over the key parameters suggests that the experimental uncertainty is low and does not significantly affect the observed trends in HIC kinetics.

Three current densities of 1.0, 4.0, and 5.5 mA/cm^2^ were applied. These current densities were selected from previous works [24] to provide a distinctive variation in the HIC kinetics. The electrolyte solution (1500 mL) consisted of 0.4 wt. % sulfuric acid in double-distilled water, with the addition of five drops of a poison solution made from 4 g of phosphorus (99.5%) dispersed in 100 mL of CS_2_ (99%). The poison solution enhances the absorption of hydrogen into the steel plate. The electrolyte solution was renewed every 72 h, with five drops (approximately 0.250 mL) of the poison solution added every 24 h, and the pH of the electrolyte solution was adjusted to 1.3 every 12 h and maintained at this value throughout the duration of the test; similarly, in the subsequent UT inspections carried out on the free surface of the steel plate, it was verified that the addition of this amount of poison solution to the specimens exposed exhibited a significantly higher number of nucleated cracks in the subsequent testing time steps, which is consistent with an increased hydrogen uptake. No direct electrochemical measurements, like hydrogen permeation or cathodic current efficiency, were performed in this study.

The growth of the area affected by HIC was monitored using an ultrasonic pulse–echo manual technique, with gel as the coupling agent, then scanning on the surface of the free side of the plate: a ¼” diameter transducer at a frequency of 20 MHz was used. Ultrasonic inspection was carried out using the C-scan technique provided by the UT equipment (NDT Systems, Nashua, NH, USA), specifically to delineate the boundaries of the area affected by HIC, and the UT inspection equipment was calibrated prior to each inspection according to the manufacturer’s proceeding. UT inspections were conducted on the area that would be in contact with the electrolyte in order to ensure the absence of cracks and to verify the condition of the material. The area affected by HIC represents a cluster of multiple individual cracks that have coalesced.

To delineate the cracked areas caused by the interconnection of cracks, the transducer was moved in zig-zag mode to locate the edges of the cracks, which were marked with a permanent ink pen on a 10 mm × 10 mm grid previously drawn on the free side of the test plate; this was performed with the DC power offline. The crack growth was monitored in this way every 24 h during the first 240 h, and then every 100 h, until no further crack growth was detected.

The criterion used to identify the corrosion boundaries affected by HIC was a 50% reduction in the ultrasonic signal amplitude, for which the −6 dB drop technique was employed. This method, widely used in the industry, enables the identification of signal boundaries with reasonable accuracy. In this case, the ±1 mm precision reported corresponds to the specifications provided by the ultrasonic inspection equipment used during the experimental test. It is important to note that this level of accuracy may vary depending on the configuration, frequency, and performance of the ultrasonic equipment and transducers employed.

**Figure 3:**
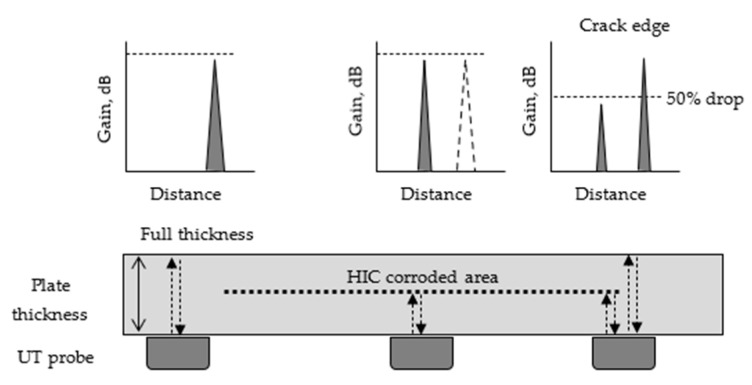
Schematic illustration of the UT 50% drop technique for detecting the HIC-corroded area.

The technique consists of detecting the HIC-corroded areas by losing the backwall reflection of the ultrasonic bean and then linearly displacing the transducer until a drop in the signal amplitude equivalent to 50% is recorded. This will indicate that the edge of the corroded area has been located. Figure 3 shows a schematic of the –6 dB drop technique; to ensure accuracy, each measurement was repeated three times, and it was verified that each measurement of the affected area was performed with the precision mentioned.

The kinetics results are reported as the total cracked area as a function of time, where the cracked area was calculated as the sum of the individual crack areas recorded after each inspection to avoid overestimation due to crack overlap; ultrasonic signals from adjacent or partially connected areas were analyzed to determine whether they represented new cracks or already-coalesced ones. In cases of overlapping regions, the affected area was counted only once, based on the outermost contour. The reason to report the cracked area instead of the crack length was because of the irregular shape of HIC cracks, which makes the determination of the crack length inconsistent.

### 2.2. Fractographic Examination

**Figure 4:**
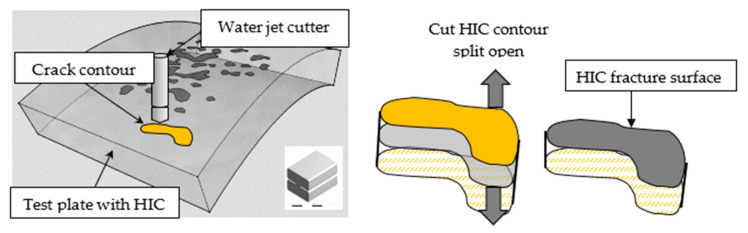
Schematic illustration of the cutting and separation of the HIC crack contours to expose the fracture surface.

To perform a fractographic examination of the surfaces fractured by HIC, a sample of the macroscopic crack contours was cut using a water jet cutting tool. This technique does not introduce mechanical damage to the fracture surfaces, as would occur with manual cutting, nor the deposition of molten material, as occurs when using oxyacetylene. The two sides of the plate were pulled apart to expose the fracture surfaces, as schematically shown in Figure 4. The examination was carried out using a JEOL 6300 scanning electron microscope (SEM), (JEOL, Tokyo, Japan) equipped with an energy-dispersive spectrometer (EDS).

### 2.3. A Theoretical Model of HIC Kinetics

**Figure 5:**
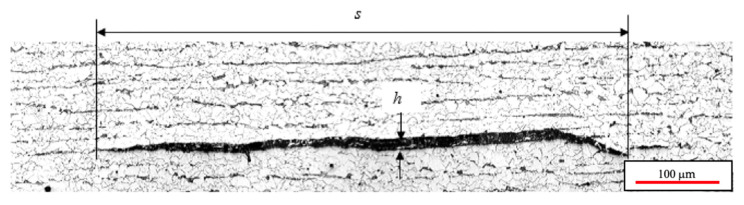
Typical shape of HIC crack in low-carbon steel. Metallographic preparation through LS section, etched using Nital solution (10% nitric acid in methanol).

Based on laboratory experiments and field inspections of HIC in low-carbon steels, it has been demonstrated that HIC, in its planar mode, initiates at nonmetallic inclusions (NMI), and the nucleated cracks have the shape of a lentil, as shown in Figure 5 [22,26]. The kinetics of HIC, on the other hand, seem to be affected by microstructural features, such as grain size, the type of second phase, and NMI characteristics [37,39], but they seem to be less dependent on mechanical properties [17,40].

Regarding the morphology of HIC, when it occurs as planar arrays of cracks, the cracks grow along the initial plane where they were nucleated, as shown in Figure 5, but eventually they can interconnect with other cracks, either on the same plane or with out-of-plane cracks, forming a stepwise array. In this case, the observed crack is subsurface, indicating that its propagation occurs parallel to the surface but at a certain depth within the material.

**Figure 6:**
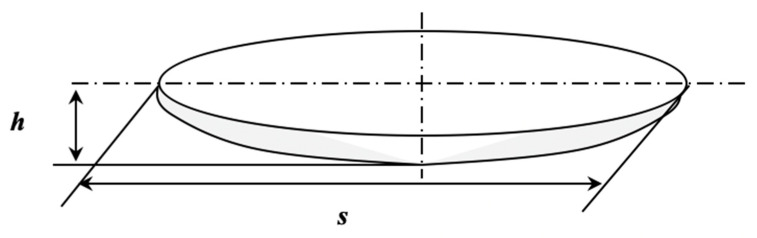
Idealized geometry of one half of an HIC crack.

In accordance with the above, Jorge Luis Gonzalez Velazquez derived a theoretical model for the kinetics of an individual internal HIC crack. The model is based on the pressure mechanism and calculates the volume change in the crack cavity per unit of time, assuming that crack growth is governed by the hydrogen flux that enters the crack cavity. The model considers an already-nucleated crack of lentil shape, Figure 6, whose rate of volume change is based on the classical solution of differential calculus, to determine the instantaneous radius of a volume that is inflated at a constant rate.

If the HIC crack cavity is a double disk, the expression used to estimate the crack volume is based on a known volumetric approximation for ellipsoidal bodies, which is commonly used for lenticular geometries [41]. Notice that Equation (1) corresponds to twice the volume of the geometry of Figure 6.

(1) $$V=\frac{2\pi }{3}\left(h^{3}+\frac{sh^{2}}{4}+\frac{s^{4}}{16h}\right)$$

where *s* is the initial crack size and *h* is the crack opening displacement normal to the crack plane, which is assumed to remain constant until the coalescence stage. This assumption is supported by multiple microscopic observations of HIC cracking, where it is consistently shown that the longitudinal growth is much greater than the axial. The key contribution of this simplification is that it reflects the observed lenticular morphology of HIC cracks, as illustrated in Figure 5. The derivation of the crack volume with respect to time is as follows:

(2) $$\frac{dV}{dt}=\frac{2\pi }{3}\left(\frac{h^{2}}{4}+\frac{s^{3}}{4h}\right)\cdot \frac{ds}{dt}={\dot{V}}_{HIC}$$

where ${\dot{V}}_{HIC}$ represents the hydrogen flux into the crack. Thus, solving for *ds*/*dt* and assuming that *s* >> *h* with (*h* = 6 μm) and integrating between the initial crack size (*s*_o_ = 20 μm) and the final crack size (*s*), for time *t* ≥ 0, an expression for crack size as a function of time is obtained.

(3) $$s=\sqrt[{4}]{\frac{24{\dot{V}}_{HIC}h}{\pi }\cdot t+s_{0}^{4}}$$

It is worth mentioning that during the crack growth stage, the s >> h ratio may be overestimated by the analytical model. However, due to the nature of HIC crack propagation, the crack length in the axial direction increases rapidly; based on a simplified analysis of the growth equation (3) and assuming the initial transverse crack opening (*h*) remains constant, we estimate that the condition *s/h* > 10 becomes valid after the first 3–5 h of simulation. Therefore, although there is some deviation at the beginning, the overall model remains robust and effectively captures the statistical evolution and coalescence behavior.

The meaning of the relationship between crack length and the fourth root of time arises from the interplay between the hydrogen flux driving crack growth and the constraints imposed by fracture mechanics. Hydrogen ingress promotes volumetric crack expansion over time; however, the material’s resistance to fracture limits the growth rate. As the crack expands, the internal pressure decreases due to the increasing volume, and the energy release rate also drops, which slows down propagation. This interaction leads to a slower growth regime in which the effective crack length follows a fourth-root-of-time dependence, representing a balance between hydrogen ingress and fracture resistance.

This model will be used later to develop an overall model of HIC growth. It is important to mention that the above model applies only for individual cracks growing in planar mode, and it does not consider interconnection or stepwise cracking.

## 3. Results

### 3.1. HIC Growth Morphology

**Figure 7:**
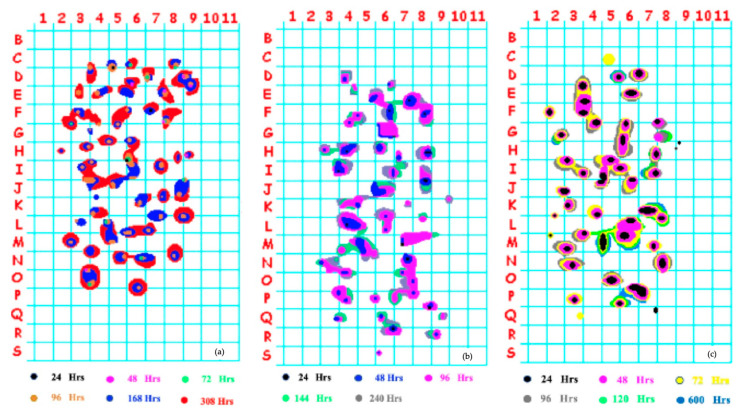
Ultrasonic mapping of the plates exposed to cathodic charging at (**a**) 1 mA/cm^2^, (**b**) 4 mA/cm^2^, (**c**) 5.5 mA/cm^2^ of DC. Grid size 10 mm × 10 mm; total area 198 cm^2^.

Figure 7a–c show the ultrasonic mappings of the plates exposed to cathodic charging at 1.0, 4.0, and 5.5 mA/cm^2^ of DC at different exposure times. The colored contours indicate the boundaries of the cracked areas on a 10 mm × 10 mm grid at different times of cathodic charging. An application software was used to draw the crack contour maps, which automatically assigned the colors of the cracked areas: that is why the colors are different in each contour map in Figure 7. The same software computed the total cracked area.

Table 3 shows the number of cracks nucleated at different times, and it does not represent the total HIC affected area; the 24 and 48 h represent the early-nucleated cracks, and the >144 corresponds to the HIC designated as late-nucleated. It can be observed that, in general, most of the individual cracks (up to 61%) appeared within the first 48 h of cathodic charging, and they are randomly dispersed in the area exposed to cathodic charging. After 144 h of cathodic charging, only 34 new cracks appeared (22%), and the interconnection of individual cracks became significant, with few new cracks continuing to appear after this time. The total number of cracks seemed to be independent of the applied current density, but the number of early-nucleated cracks (up to 24 h) was dependent on the applied current density.

### 3.2. HIC Kinetics

To determine the kinetics of HIC, three independent tests were conducted (A2, A3, A4), each one consisting of steel plates exposed to different current densities of 1.0 mA/cm^2^, 4.0 mA/cm^2^, and 5.5 mA/cm^2^, under the following considerations:

(a) Cracks of near-circular shape nucleated within the first 24 h of cathodic charging.
(b) Cracks growing individually or with interconnection only at the moment of inspection.
(c) Irregularly shaped cracks or very elongated cracks were not considered.

**Figure 8:**
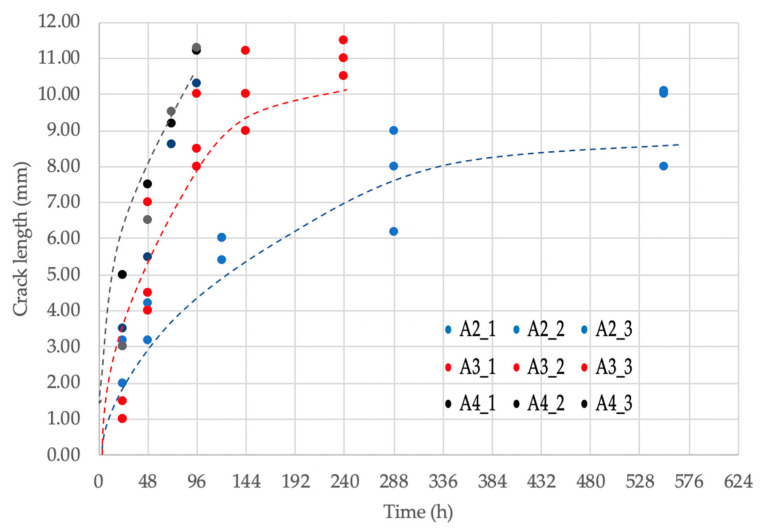
Experimental results pertaining to HIC kinetics of individual cracks in steel plates exposed to cathodic charging. A2: i = 1.0 mA/cm^2^. A3: i = 4.0 mA/cm^2^. A4: i = 5.5 mA/cm^2^.

The experimental results regarding the HIC growth rate are presented in Figure 8. The dotted lines represent a regression fit that was applied to the upper crack size values observed at each current density level, and the individual data points represent the average values of each one of the three test repetitions. The overall trend of the data shows a nearly linear behavior in the initial hours of cathodic charging, corresponding to the growth of individual cracks. However, after 72, 144, and 336 h for 5.5, 4.0, and 1.0 mA/cm^2^ respectively, the trend deviates from linearity. Although the nucleation of new cracks virtually stops after 240 h, some individual cracks continue to grow and eventually interconnect with nearby cracks. This coalescence process contributes to the slight increase in the total cracked area, without necessarily implying the formation of new cracks.

### 3.3. Fractographic Examination

**Figure 9:**
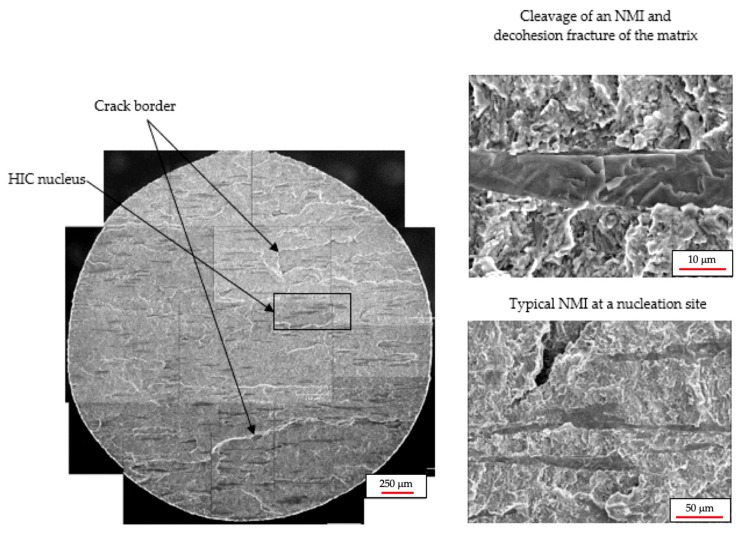
Fracture surface generated by HIC by cathodic charging at 1 mA/cm^2^.

The fracture surface of an initiation site of a typical HIC crack as viewed in the scanning electron microscope (SEM) is shown in Figure 9. It can be observed that there is a relatively high density of inclusions in the fracture plane, averaging 45 inclusions per mm^2^; this value was obtained using an automated image analysis system coupled to an optical microscope. The metallographic samples were prepared and examined at 100× magnification. Inclusion counting was performed using calibrated image analysis software, which automatically detects and quantifies nonmetallic inclusions within multiple fields of view. The results are relative to the observed area, and the final value corresponds to the average number of inclusions per square millimeter. A closer examination of the sites identified as nuclei of the initial HIC cracks indicates that these sites are clusters of MnS inclusions of more than 500 µm in length. At a higher magnification, it becomes evident that the crack growth mechanism is brittle, primarily due to decohesion of pearlite-ferrite interfaces. It is worth mentioning that the MnS inclusions show cleavage fractures, and there are some relief marks on the fracture surface that correspond to the borders of individual HIC cracks.

**Figure 10:**
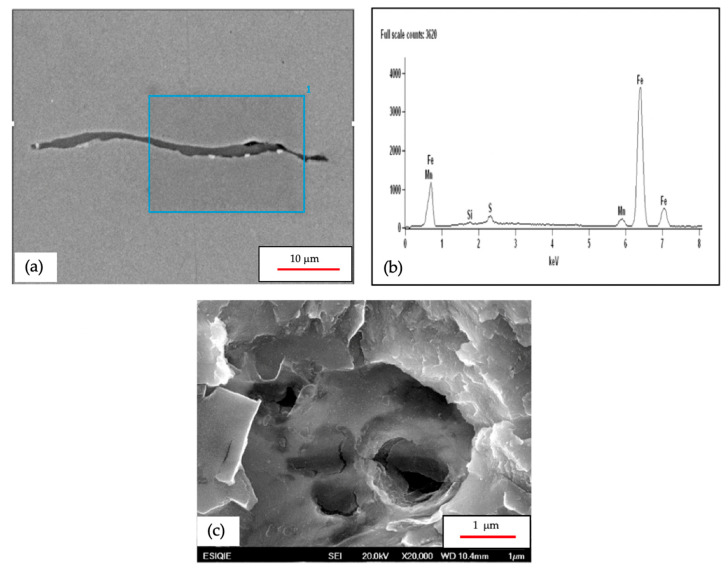
(**a**) SEM image showing a typical manganese sulfide (MnS) inclusion, located at a subsurface region of the steel plate. (**b**) Energy-dispersive X-ray spectroscopy (EDXS) image was obtained from the region highlighted in blue Figure 10a confirming the chemical composition of the inclusion, with prominent Mn and S peaks. (**c**) SEM micrograph of the crack cavity near the crack tip.

Figure 10 shows a high-resolution SEM and an EDXS (JEOL, Tokyo, Japan) image of one of these MnS inclusions and its cavity, with brittle-like cracks. The inside can be seen at the tip of the MnS inclusion, indicating that the HIC crack was initiated by the burst caused by the fugacity of the hydrogen gas trapped by the MnS inclusion [21], and it appears that after the MnS inclusion broke apart, the metal matrix began to fracture around the MnS inclusion. As previously mentioned, the MnS inclusion predominantly exhibits cleavage or quasi-cleavage fractures. No quantitative analysis of the fracture modes was performed across multiple fields of view; therefore, the current interpretation is based on qualitative observation.

### 3.4. Computational Modeling of HIC Kinetics

According to the experimental observations, the initial HIC cracks were found to nucleate predominantly at subsurface regions, typically in NMIs, such as MnS; in the computational model, the initial crack sites were randomly distributed within the 2D domain to reflect the random spatial distribution of favorable inclusions acting as hydrogen traps; the model does not currently resolve depth, and it assumes that all nucleation occurs in a representative mid-thickness plane of the material.

The first step in developing the model of HIC growth was to determine the type of spatial distribution of the initial HIC cracks recorded in the cathodic charging experiments. To answer this question, the Ripley’s K-function was applied to the HIC initiation sites, represented by the black contours after 24 h of cathodic charging in Figure 7.

The Ripley’s *K* test requires the calculation of the $\hat{K}$ value of the observed dispersion pattern, which is compared against the *K* value of the theoretical function for a Poisson pattern of the same intensity [42]. Ripley’s *K*-function is defined as follows:

(4) $$K\left(r\right)=\lambda^{-l}$$

where *λ* is the density of initiation sites per unit area, and *l* is the average number of sites within a radius, *r*, around any other site. The K-function can be defined by *λK*(*r*), which represents the average number of initiation sites within a circle around a “typical” site in the pattern and describes the characteristics of the point process (sites) across multiple scales, depending on the different values of *r* considered in the analysis. The equations to estimate *λ* and *K*(*r*) are as follows:

(5) $$\hat{\lambda }=\frac{N}{A}$$

(6) $$\hat{K}\left(r\right)=\frac{1}{\hat{\lambda }}\frac{1}{N}\sum_{i=1}^{N}\sum_{j\neq i}^{N}l\left(d_{ij}<r\right)$$

where *N* is the number of sites in the dispersion pattern, *A* is the study area, and *l*(*d_ij_* < *r*) is the indicator function that takes a value of 1.0 if the distance between points i and j is less than *r* and zero if otherwise. In other words, it determines whether the evaluated point or site lies within or outside the area of a circle with radius *r*.

The assessment criteria to determine if the point distribution is random are as follows [42]:

1. If the point distribution is random, the *K* curve deviates little from π*r*^2^, and the *K* curve remains close to the reference value, ${\pi r}^{2}$, for all radii, r.
2. If the point distribution is regular, $\hat{K}\left(r\right)$ < $K\left(r\right)$. Because the points are repulsive, they have fewer neighbors on average in a radius than they would have based on the assumption of a random distribution of points.
3. In the case of an aggregated distribution, there are more points in a radius around the points than the expected number under a random distribution: consequently, the points attract each other and $\hat{K}\left(r\right)$ > $K\left(r\right)$.

Applying this method to the distributions of the initial HIC cracks displayed in Figure 7, the result is that the spatial distribution of the initial HIC cracks is completely random; therefore, the initial distribution of HIC cracks in the modeling code was implemented using a Poisson distribution, as the experimental statistical analysis indicated that this distribution best represents the spatial arrangement of the initial HIC nucleation sites observed in the material.

The numerical simulation was carried out using the high-level programming language Python™ 3.11.3 to simulate the nucleation, growth, and interconnection of HIC cracks in steel exposed to a hydrogen-charging environment; the simulation was conducted up to the first 400 h for comparison with the laboratory experimental results.

The mathematical procedure applied to develop the HIC growth model is as follows:

1. Poisson Process: The process of generating initial points is based on a Poisson distribution to simulate the location of the initially formed HIC cracks in a completely random distribution of individual cracks that grow independently of each other, using a two dimensional rectangular domain of 110 × 180 arbitrary units, corresponding to 11 cm × 18 cm in physical dimensions. This domain represents the exposed section of the steel in contact with the electrochemical solution; the nuclei were randomly distributed within this domain, and open boundary conditions were assumed to allow cracks to evolve naturally without artificially constraining their growth near the domain edges. Throughout the simulation, no significant clustering or accumulation of cracks near the boundaries was observed. This is attributed to the use of a homogeneous Poisson spatial distribution of nucleation sites and the statistical nature of crack growth and coalescence, which together promote a uniform evolution of damage across the domain. Crack growth was too confined within the domain limits, and cracks reaching the edges were not allowed to propagate beyond them. The Poisson distribution is defined as follows [43]:
  (7) $$f\left(x\right)=P\left(X=x\right)=\frac{e^{-\lambda }\lambda^{x}}{x!}$$
  Where *P* (*X* = *x*) is the probability of *x* events occurring, *x* is a non-negative integer, *X* is a discrete random variable, λ is a positive constant, and *x!* is the factorial of *x*.
2. Ellipsoidal Geometry: These cracks are modeled as ellipses in the plane and are mathematically defined by the following equation:
  (8) $$\frac{{\left(x-h\right)}^{2}}{a^{2}}+\;\frac{{\left(y-k\right)}^{2}}{b^{2}}\;=1$$
  where (*h*, *k*) are the coordinates of the center of the ellipse, *a* is the major axis of the ellipse, and *b* is the minor axis. Each generated point can be the center of an ellipse, but the initially defined semi-axes can change over time due to the hydrogen intake.
3. Interconnection: The interconnection is modeled as the overlap area among multiple ellipses by using the Monte Carlo technique [44] to simulate random points within the domain and count how many fall inside any ellipse, providing a statistical estimate of the overlap area. The Monte Carlo method introduces a degree of statistical uncertainty that depends on the number of sampled points; larger sample sizes reduce variability in the estimation of the overlapped area.
4. Time Tracking: The code iterates in time increments to simulate the temporal evolution of the total crack area by HIC.

It is important to mention that at this stage, the integration of the theoretical model, the spatial Poisson distribution, and the Monte Carlo-based algorithm was designed to statistically capture the evolution and coalescence of HIC damage over the time, rather than to resolve local stress redistribution or crack–crack interactions prior to intersection; therefore, the model does not incorporate elastic or plastic stress fields around individual cracks and, thus, does not account for the influence of neighboring stress fields on the growth direction or rate.

By using the model proposed by Gonzalez, HIC growth was simulated considering 1.0, 4.0, and 5.5 mA/cm^2^ values of cathodic charging current density, which correspond to hydrogen fluxes of 0.08, 1.03, and 1.59 mm^3^/seg, calculated by the Faraday’s Law. The initial crack size at 24 h of hydrogen charging was 20 µm in the longitudinal direction and 6 µm in the transverse direction; the model was run for up to 400 h.

**Figure 11:**
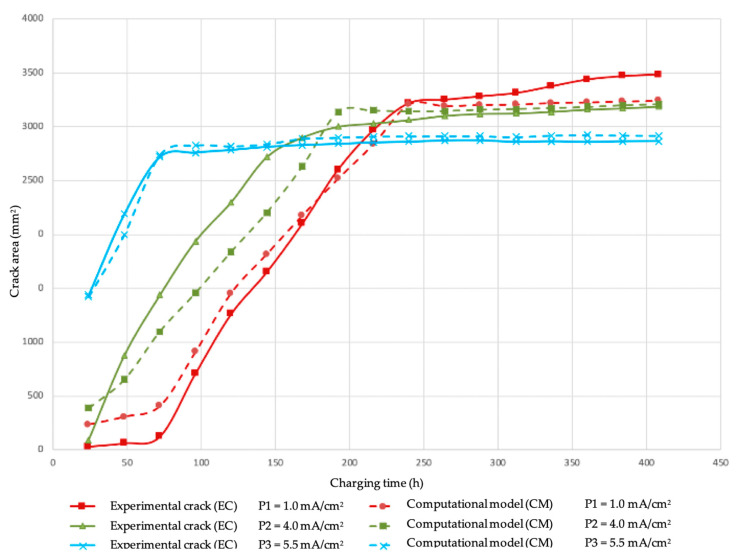
Crack growth behavior: experimental vs. computational model.

Figure 11 shows the experimental results versus the computational simulation. It is observed that the results agree fairly well for the 1.0 and 5,5 mA/cm^2^ applied current densities, but for the 4.0 mA/cm^2^ case, during the individual crack growth stage (Stage I), the simulation results exceed the experimental trend. The adjusted R^2^ correlation values are 0.99, 0.93, and 0.93, respectively, showing good convergence between the simulated and the experimental results.

## 4. Discussion

The experimental results shown in Table 3 indicate that approximately 61% of HIC cracks nucleate within the first 48 h of hydrogen charging, with the number of early-nucleated cracks (charging time ≤ 24 h) being proportional to the applied current density. Notably, the maximum number of nucleated HIC sites per unit area, calculated as 0.0028 cracks/cm^2^ based on the data of Table 3, is significantly lower than the number of nonmetallic inclusions (NMIs), reported as 45 inclusions/mm^2^. This suggests that HIC initiates at only a small fraction of NMIs that have favorable characteristics to act as hydrogen traps and start the HIC process. A more detailed study of the characteristics that make NMIs become HIC nucleating sites has been presented in previously published papers [24,25,37].

Regarding the morphology of HIC, the results indicate that the distribution of the nucleation sites appears to be random, resulting in a random distribution of individual HIC cracks during Stage I. The morphology in Stage II, characterized by the interconnection of cracks, is also predominantly random. However, if the distribution of NMIs is not random, for any reason, the kinetics of HIC will be certainly modified with respect to what was observed here. For example, if there is a concentration of inclusions in specific regions, the coalescence of neighboring cracks will be facilitated, and that will shorten the transition to Stage II. Furthermore, the cracks will align in bands, resulting in a very different HIC crack morphology.

An important experimental observation of this work is that the kinetics of HIC and the number of early-nucleated cracks are directly proportional to the applied current density in the cathodic charging experiment; this is clear since the current density determines the flux of hydrogen into the steel plate. At first, the rationale of this behavior is because a higher applied current density generates a greater amount of atomic hydrogen, which diffuses into the steel plate, increasing the likelihood of activating the nucleation sites at suitable NMIs. However, in Stage I of HIC, once a crack is formed, the driving force for growth is the pressure buildup of hydrogen in the crack cavity, so with multiple cracks competing for hydrogen intake, the number of activated nucleation sites decreases, leading to a reduction in the rate of formation of new cracks. Further, when all of the available nucleation sites are activated, as occurred after approximately 200 h of cathodic charging, as shown in Figure 7, the nucleation of new cracks becomes nearly zero, and the growth of the cracked area is primarily due to the coalescence of the previously formed cracks.

Another observation is that at applied current densities of 4.0 mA/cm^2^ or less, there is a delay in the nucleation of HIC, as can be seen in Figure 11. This condition may be attributed to the transitional nature of corrosion product layer formation under intermediate current densities, where a semi-protected layer may form that neither fully blocks hydrogen entry nor remains entirely unstable [45,46]. This leads to a more complex and time-dependent behavior that is difficult to represent with a single fixed correction factor; as a result, the adjustment applied in the model may not fully account for the dynamic evolution of the corrosion product layer, contributing to the observed deviation. However, this corrosion layer becomes more porous and mechanically unstable, so after about 24 h it loses its ability to impede hydrogen diffusion and the kinetics of HIC is fully activated. It is important to mention that at applied current densities of 5.5 mA/cm^2^ the activation of HIC is almost immediate; this can be due to the removal of layer of corrosion products by the presence of film defects such as pores and grain boundaries [46] and because the hydrogen flux is sufficient to introduce enough hydrogen to activate the available nuclei.

Another important result is that the HIC growth rate experimentally measured and calculated by the model was nearly constant in each stage for each applied current density, as seen in Figure 11. This behavior can be explained by combining the observed fracture mechanism (described in Section 3.3) and the pressure mechanism described in Section 2.3. As mentioned, the HIC cracks grow in a planar mode, with very little expansion in the thickness direction, perpendicular to the cracking plane. Since the fracture mechanism of both crack nucleation and growth is brittle and is essentially the same for the initiation and growth of HIC, the crack growth rate depends on reaching the internal pressure in the crack to overcome the cohesive strength of the crack plane, which it is reasonable to believe is constant and dependent on the microstructure and the hydrogen concentration in the lattice for each particular steel composition and microstructure. The specific role of the microstructure and the mechanical properties of the steel plate on the kinetics of HIC is an open field for further research. Some of the results obtained so far by our research group can be seen in the references by Szpunar, Gonzalez, and Entezari listed in this paper.

To introduce into the computational code the delay of the start of HIC, attributed to the formation of corrosion layers, a reduction of the hydrogen flux in the initial hours of HIC was artificially introduced, being 2% in the P1 experiment (i = 1 mA/cm^2^), 15% in the P2 experiment (i = 4 mA/cm^2^), and no reduction in the P3 experiment (i = 5.5 mA/cm^2^); this because there was no delay in this case. The percentages (2% and 15%) used to simulate the delay in the onset of hydrogen-induced cracking (HIC) due to the presence of corrosion product layers were manually adjusted. These values were not derived from direct electrochemical measurements but were empirically calibrated to achieve a better match between the simulated crack growth curves and the experimental observations. The total applied current density was reset at the nominally applied value after 75 h of simulation. Although the quantitative characterization of the iron sulfide layers was not the primary objective of this study, the incorporation of this dynamic delay significantly improved the agreement between the simulated results and experimental observations.

The model proposed here showed the capability to correctly simulate the kinetics and morphology of HIC nucleation and growth and represents an important first step towards developing a HIC life prediction algorithm for in-service process piping and pieces of equipment made of low-carbon steel that are exposed to hydrogen-charging environments, which is lacking in the industry.

Although the HIC cracks are inherently three-dimensional in nature, projecting them as two-dimensional features could introduce some level of simplification. However, the cracks observed experimentally in this work exhibited predominantly planar growth, with very limited propagation through the thickness direction; this is consistent with previous studies [1,10,21] reporting that HIC tends to occur along specific planes (parallel to the rolling direction or mid-thickness). Therefore, the 2D modeling approach used is a reasonable simplification that allows for effective statistical analysis of crack nucleation, growth, and coalescence, and while it may not capture the full 3D morphology of each individual crack, it adequately reflects the area evolution and interaction patterns that are critical to understanding the kinetics of HIC.

In this study, HIC nucleation was consistently observed in close association with elongated manganese sulfide inclusions, as confirmed by EDXS analysis and metallographic examination. Although recent works, such as that of Entezari et al. [25], have proposed systematic classifications of nonmetallic inclusions to assess their effectiveness as HIC nucleation sites, this approach was not fully applied here. The reason is that the tested steel exhibited a uniform population of MnS-based inclusions, with no significant variations in the type or morphology observed. Therefore, the implementation of a broader classification scheme was deemed unnecessary for this case. Nevertheless, for future studies involving steels with more heterogeneous NMI populations, the incorporation of detailed classification frameworks may offer valuable insights into microstructural susceptibility to HIC and enhance the predictive accuracy of computational models.

The integration of future versions of the computational model with Non-Destructive Testing (NDT) techniques—such as industrial ultrasound, acoustic emission, or infrared thermography—can be achieved through various approaches that directly link numerical predictions with measurable physical responses. Specifically, for industrial ultrasound, the model could be extended to simulate theoretical acoustic responses. Based on the simulated geometry, size, and statistical distribution of cracks, it would be possible to estimate parameters, such as time-of-flight, signal attenuation, and echo amplitude, that are commonly measured in ultrasonic testing. This would allow for the establishment of more accurate detection thresholds and the calibration of critical parameters, such as crack growth rates, nucleation site distributions, and kinetic delays, associated with corrosion product layer formation. Additionally, the data generated by the model could be used to build synthetic datasets for training artificial intelligence algorithms aimed at the automatic interpretation of NDT signals acquired in the field.

Based on what has been presented, the originality of this research lies in the way that statistical and numerical methods were integrated to simulate the complete statistical evolution of the HIC in a unified and time-dependent framework using the stochastic spatial distribution of initial nucleation sites; a simplified, pressure-driven growth law based on hydrogen flux; dynamic adjustment of hydrogen entry to account for corrosion product layers (which is rarely considered explicitly in similar models); and a Monte Carlo-based algorithm to quantify the effective cracked area and track coalescence events statistically over time; this contrasts with previous studies [25,39] that have largely focused on metallurgical parameters, such as second-phase distributions, inclusion content, or morphology, or purely deterministic crack growth formulations.

Finally, it is yet necessary to incorporate metallurgical characteristics, such as grain size and the type of secondary phases (such as pearlite, bainite, or martensite), into the modeling algorithm, since these features directly influence the hydrogen diffusion behavior and affect the in-plane fracture strength of the microstructure, thereby affecting the resistance to HIC propagation. Similarly, the nature of NMIs shall be also incorporated into the model to allow for more realistic simulations of different material conditions or specifications. Additionally, a future objective is to perform electrochemical characterization of the corrosion product layers in order to allow more accurate adjustments to the HIC kinetics by quantifying the delay effect associated with the formation of such layers.

## 5. Conclusions

1. The model proposed in this study successfully simulated the kinetics and morphology of hydrogen-induced cracking (HIC) nucleation and growth in a low-carbon steel plate under static loading conditions. It does not currently account for cyclic stresses or temperature fluctuations. The model incorporates key factors, such as the spatial distribution of HIC nucleation sites, the hydrogen influx represented by the applied current density, and experimentally observed features, like the delayed onset of HIC. This framework offers a valuable tool for understanding HIC evolution by varying input parameters and analyzing the resulting total cracked area and the final morphology of HIC-damaged regions in low-carbon steel plates. It is considered an important first step toward developing a predictive algorithm for estimating the remaining service life of process piping and equipment made of low-carbon steel exposed to hydrogen-charging environments. Future improvements to the model will aim to incorporate microstructural parameters, such as grain size and phase distributions, local stress fields, cyclic loading conditions, and steel samples with different fracture toughness levels, in order to evaluate how these variables affect HIC kinetics.
2. The results of investigations into the kinetics and morphology of hydrogen-induced cracking (HIC) in low-carbon steel, determined after the cathodic charging of steel plates, indicate that the number of nucleated cracks and the kinetics of HIC are proportional to the applied current density, primarily because it determines the hydrogen influx. Furthermore, it was observed that the activation of HIC nuclei sites is also proportional to the applied current density in the cathodic charging experiment. It was found that HIC initiates at only a small fraction of NMIs that have favorable characteristics to act as hydrogen traps and start the HIC process, and their spatial distribution is a key factor in the overall kinetics and morphology of HIC.
3. It was observed that the HIC process is divided into two stages: Stage I, nucleation and growth of individual cracks, and Stage II, interconnection of cracks. The growth rate is not constant in each stage, being higher in Stage I and very low in Stage II. This behavior was explained by the combined effects of the fracture mechanism (quasi-cleavage) and the pressure mechanism, which suggest that the nucleation and growth of HIC depend on reaching the internal pressure in the crack that is sufficient to overcome the cohesive strength of the crack plane. This strength is constant and dependent on the microstructure and the hydrogen concentration in the lattice for each particular steel composition and microstructure.

## Figures and Tables

**Table 1:** The chemical composition of the API 5L X52 steel plates used in the experiment, in wt%.

C 0.106	Si 0.184	Mn 0.844	P 0.006	S 0.026	Cu 0.270	Al 0.025	Cr 0.041
Mo 0.0007	Ni 0.019	V 0.010	Ti 0.004	Nb 0.030	W 0.013	B 0.001	Fe 98.4

**Table 2:** Microstructural characterization of the steel plates used in the experimental study.

Sample Direction	Area Inclusions %	ASTM Inclusion Type	Ferrite %	Pearlite %
LS	0.38	A	93.77	6.23
TS	0.39	A	92.78	7.22

**Table 3:** Count of HIC nuclei.

Current/Time	24 h	48 h	>144 h	Total
1 mA/cm^2^	3	6	32	56
4 mA/cm^2^	13	26	1	50
5.5 mA/cm^2^	43	1	0	44
Subtotal	59	33	34	150

## Data Availability

The original contributions presented in this study are included in the article. Further inquiries can be directed to the corresponding author.
